# Treatment Outcomes of Lobular Granulomatous Mastitis: Impact of Hyperprolactinemia, Diabetes, and Erythema Nodosum—Insights From a 7‐Year Cohort Study

**DOI:** 10.1155/tbj/2672157

**Published:** 2026-03-23

**Authors:** Mina AkbariRad, Fereshte Sheybani, Abdollah Firoozi, Samaneh Sajjadi, Maryam Emadzadeh, Marzieh Kazerani, Sajad Ataei Azimi, Mahdieh Mottaghi

**Affiliations:** ^1^ Department of Internal Medicine, Faculty of Medicine, Mashhad University of Medical Sciences, Mashhad, Iran, mums.ac.ir; ^2^ Department of Infectious Diseases and Tropical Medicine, Faculty of Medicine, Mashhad University of Medical Sciences, Mashhad, Iran, mums.ac.ir; ^3^ Mashhad University of Medical Sciences, Mashhad, Iran, mums.ac.ir; ^4^ Clinical Research Development Unit, Ghaem Hospital, Mashhad University of Medical Sciences, Mashhad, Iran, mums.ac.ir; ^5^ Department of Infectious and Tropical Diseases, School of Medicine, Islamic Azad University, Mashhad Branch, Mashhad, Iran, azad.ac.ir; ^6^ Department of Hematology and Oncology, Faculty of Medicine, Mashhad University of Medical Sciences, Mashhad, Iran, mums.ac.ir

**Keywords:** diagnosis, granulomatous mastitis, mastitis, treatment outcome

## Abstract

**Background:**

This study presents our observations on the management and follow‐up of patients diagnosed with lobular granulomatous mastitis (LGM) in a cohort study. Additionally, characteristics associated with a longer disease course, as well as treatment challenges in patients with erythema nodosum, diabetes, and hyperprolactinemia, would be discussed.

**Methods:**

From 2015 to 2021, a total of 246 consecutive LGM patients referred to the internal medicine clinic of Ghaem teaching hospital, Mashhad, Iran, were enrolled. Regular assessments were conducted every 3 months until complete symptom resolution. Treatment responses were categorized into five groups: complete resolution, incomplete resolution, resolution with subsequent relapse, no significant improvement, and treatment cessation, with all data meticulously recorded. Telephone follow‐ups were conducted with all patients at the end of the study in December 2022.

**Results:**

A total of 156 episodes were analyzed. Prednisone was administered to 136 patients, while oral methotrexate (MTX) was prescribed to 48 cases. The median age of the cohort was 33 years (interquartile range [IQR], 29–38). Of those on prednisone, 57 (41.9%) achieved complete resolution, with 15 (11%) experiencing subsequent relapse, 33 (24.3%) showing no significant improvement, and 31 (19.9%) discontinuing treatment. Among the MTX recipients, 23 (47.9%) achieved complete resolution, while one showed incomplete resolution. Over the median follow‐up of five years (IQR, 4–6), 139 (89.1%) reported complete resolution, nine (5.8%) showed incomplete resolution, and 8 (5.1%) remained symptomatic. The median disease duration was 18 months (IQR, 7–36). Abscess formation during treatment correlated with prolonged disease duration (*p* < 0.04), and higher plasma prolactin levels were associated with extended disease duration (*p* = 0.001). However, the disease course did not significantly differ in diabetic cases or those with erythema nodosum compared to others.

**Conclusions:**

Although more than half of LGM patients experienced no significant improvement, recurrence, or discontinued treatment on prednisone or MTX, however, over a median follow‐up of 5 years, approximately 90% of LGM patients achieved complete resolution within a median course of 18 months. The presence of abscesses during treatment and elevated plasma prolactin levels was linked to longer disease duration.

## 1. Background

Lobular granulomatous mastitis (LGM) manifests as a benign inflammatory condition characterized by the formation of non‐necrotizing granulomas within the breast tissue. While LGM predominantly affects women who have given birth and have a history of breastfeeding [1], cases have also been documented in nulliparous women and men [2]. Recent reports have described atypical presentations of LGM, highlighting diagnostic challenges and heterogeneous treatment responses that underscore the need for individualized, patient‐centered management strategies [3].

Several studies have suggested an autoimmune mechanism underlying the development of LGM [4, 5]. Additionally, environmental factors such as trauma, hormonal changes, metabolic fluctuations, leakage of lactational secretions, and infection with Corynebacterium species have been proposed as potential triggers for LGM [5–8].

While LGM may resolve on its own, especially in mild instances, it often presents significant debilitation [9–11], warranting treatment for symptom management and cosmetic concerns. Corticosteroids (CSs) have demonstrated efficacy in treating LGM, although the optimal dosage and duration remain debated [1, 12, 13]. Other anti‐inflammatory medications, such as methotrexate (MTX), azathioprine, and mycophenolate mofetil, have also been utilized in LGM treatment [14–16].

In this cohort study, we describe the demographic characteristics, clinical presentations, treatment strategies, and clinical outcomes of 156 patients diagnosed with LGM.

## 2. Methods

### 2.1. Patients

This prospective cohort study enrolled patients diagnosed with pathologically confirmed LGM who were referred to the internal medicine clinic of Ghaem Teaching Hospital in Mashhad, Iran, between January 2015 and December 2021. To exclude secondary etiologies, tuberculin skin tests, bacterial, mycobacterial, and fungal staining and cultures, as well as polymerase chain reaction (PCR) testing for *Mycobacterium tuberculosis*, were conducted. Plasma levels of angiotensin‐converting enzyme (ACE), prolactin, fasting blood sugar (FBS), and erythrocyte sedimentation rate (ESR) were measured for all patients.

Individuals with follow‐up periods of less than 1 year, those diagnosed with *tuberculosis* mastitis, and male patients were excluded from the study. Data regarding patient history, presenting symptoms and signs, laboratory and imaging results, treatment regimens, and outcomes were collected to achieve the following objectives: (1) assess treatment management and outcomes in LGM patients, (2) evaluate the impact of LGM in individuals with diabetes, hyperprolactinemia, and erythema nodosum (EN), and (3) identify characteristics associated with a prolonged disease course.

### 2.2. Ethical Considerations

This study was approved by the Ethics Committee of Mashhad University of Medical Sciences under project numbers 4010734 and the ethics codes of IR.MUMS.MEDICAL.REC.1401.609. All investigations were consistent with the Declaration of Helsinki. This study was a prospective registry study, and written informed consent was obtained from all participants at the time of diagnosis. During telephone follow‐up, patients were reassured that their information would remain confidential and would be used only for research purposes to improve the management of similar patients.

### 2.3. Treatment Approaches

**FIGURE 1:**
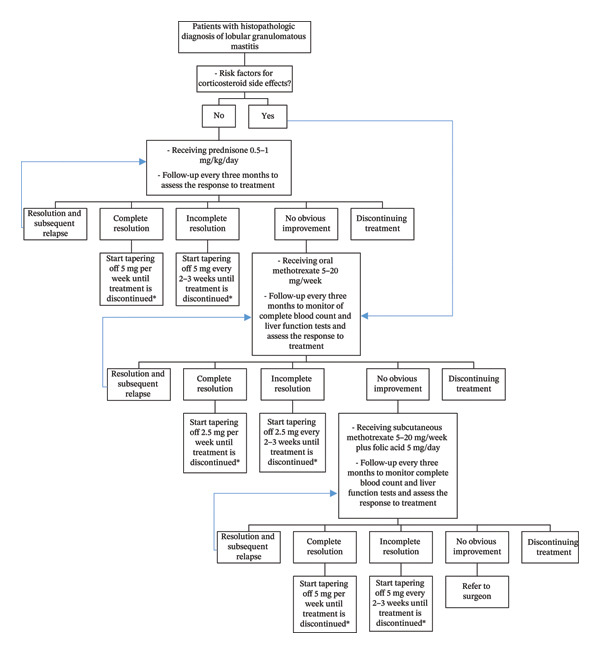
The management of patients with LGM from the beginning of the study until the complete resolution of symptoms.

All patients received detailed information regarding the disease’s prolonged and self‐limiting nature, which typically resolves gradually over time. Treatment modalities included the administration of prednisone, MTX, or referral to a surgeon (Figure 1).

Prednisone was considered the standard first‐line treatment approach for patients with newly diagnosed LGM in our cohort. However, not all patients were eligible to receive CSs due to pregnancy, patient refusal, significant risk factors for CS‐related adverse effects, or mild disease suitable for expectant management. Prednisone therapy commenced at a dosage ranging from 0.5 to 1 mg/kg/day, adjusted according to the severity of symptoms, and typically continued for 2–4 weeks. Subsequently, patients underwent regular physical examinations every 3 months following the initiation of treatment. Treatment outcomes were classified into five distinct categories: complete resolution, partial resolution, recurrence, persistence, and treatment cessation (Table 1).

**TABLE 1:** Classification of “response to treatment” in patients with lobular granulomatous mastitis.

Complete resolution	Disappearance of breast mass(es) upon physical examination and substantial reduction in inflammatory signs and symptoms.
Partial resolution	Significant decrease in breast mass(es) size upon physical examination or improvement in inflammatory signs and symptoms, but not complete resolution.
Recurrence	Initial disappearance of breast mass(es) upon physical examination and notable reduction in inflammatory signs and symptoms, followed by recurrence within at least 1 month.
Persistence	Continued presence of signs and symptoms without significant improvement.
Treatment cessation	Decision to discontinue management.

In cases where symptoms completely resolved, the dosage of prednisone was gradually tapered by 5 mg per week. Conversely, for patients with incomplete resolution, prednisone was tapered more gradually at a rate of 5 mg every 2–3 weeks. After achieving complete symptom resolution, patients ceased follow‐up appointments but were advised to return to the clinic if signs or symptoms recurred. If symptoms reappeared during or after the tapering of CSs, another course of prednisone was prescribed. Those who discontinued treatment were monitored via telephone follow‐ups.

For individuals showing no significant improvement on CSs, prednisone was tapered off and oral MTX was initiated at a dosage of 5–20 mg per week, adjusted according to symptom severity. All patients on MTX received daily folic acid supplementation at a dose of 5 mg. Follow‐up appointments were scheduled every 3 months, during which regular check‐ups for complete blood count (CBC) and liver function enzymes were conducted. Treatment response was evaluated using previously outlined criteria. Upon resolution of symptoms, MTX dosage was gradually reduced by 2.5 mg per week, with patients advised to return to the clinic only if symptoms recurred. Patients who did not respond to oral MTX received subcutaneous MTX at the same dosage range and continued follow‐up as instructed.

In cases of persistent severe inflammatory symptoms despite treatment with prednisone and/or MTX, surgical excision of the lesion (lumpectomy) was performed. Patients experiencing abscess formation underwent incision and drainage.

Telephone follow‐up was conducted at the end of the follow‐up period in December 2022. Patients were queried about their symptoms, any episodes of relapse, treatment side effects, and other types of treatments they might have pursued. If patients reported mastitis symptoms or side effects, they were advised to return to the clinic for further clinical assessment. The duration of the disease was defined as the time interval between symptom onset and complete recovery without subsequent relapse.

### 2.4. Statistics and Sample Size

Continuous data were presented as medians with interquartile range [IQR] (25th–75th percentile), while categorical variables were expressed as frequencies and percentages. Normal distribution was evaluated using the one‐sample Kolmogorov–Smirnov test. For continuous variables with non‐normal distribution, the Mann–Whitney *U*‐test was employed for comparison, whereas Fisher’s exact test and chi‐square tests were utilized for categorical variables, as appropriate. A *p* value < 0.05 was considered statistically significant. Correlation analysis was conducted using the Spearman test. The sampling method employed was consecutive, with all eligible patients included in the study.

## 3. Results

Out of the initial 246 patients diagnosed with LGM and included in our cohort, 67 were excluded due to incomplete documentation, 18 patients missed subsequent follow‐ups, four were diagnosed with *tuberculosis* mastitis, and one male patient was excluded. Consequently, our analysis focused on 156 female patients with idiopathic LGM.

### 3.1. Patients

The median age of the cohort was 33 years (IQR, 29–38), with 96.2% of patients being married. The median number of children was two (IQR, 1–3), with eight cases being nulliparous. Fifteen patients (9.6%) were using hormonal contraception, and five (3.2%) reported a history of trauma to the affected breast. The median interval between the onset of symptoms and referral to our clinic was 90 days (IQR, 60–180). Twenty‐one patients (13.5%) had experienced previous episodes of IGM. Clinical findings at the initial appointment included breast mass in 114 cases (73.1%), pain/tenderness in 102 cases (65.4%), erythema in 56 cases (35.9%), nipple retraction in 41 cases (26.3%), discharge in 42 cases (26.9%), and axillary lymphadenopathy in 27 cases (17.3%). The discharge was from a sinus tract to the lesion surface in 27 patients, from the nipple in 10 cases, and from the biopsy site in five patients. Five cases reported purulent discharge. Symptoms were unilateral in 126 patients (80.8%), while subsequent involvement of the second breast occurred in 30 patients (19.2%). Ultrasound examination revealed a mean mass size of 2.6 cm, with 18 cases (11.5%) having a breast mass larger than 5 cm. Extra‐mammary manifestations included fever at disease onset in 11 patients (7.1%), EN in 8 patients (5.1%), and peripheral arthritis in 14 patients (9%).

At the initial appointment, 43 cases (28.1%) were diagnosed with a breast abscess, while 50 patients (32.1%) developed an abscess from the breast mass during treatment. The median plasma levels of prolactin in nonpregnant patients, ACE, and ESR were 12.7 (IQR, 7.9–21) ng/mL, 38 (IQR, 26–51.2) U/L, and 25 (IQR, 13.75–45.25) mm/h, respectively.

### 3.2. Management

In total, prednisone was administered as the primary treatment to 136 patients (87.2%), ranging from 3 to 60 months. Thirteen cases (8.3%) had previously undergone surgery for excision of the breast mass, while 12 patients (7.7%) had received incision and drainage for the lesion. Among those treated with CSs, complete resolution was observed in 57 patients (41.9%), 15 patients (11%) experienced resolution followed by relapse, and 33 patients (24.3%) showed no significant improvement. Thirty‐one cases (22.8%) discontinued treatment, with 21 attributed to poor compliance, eight due to adverse medication effects, and two due to pregnancy.

Follow‐up calls with patients who discontinued prednisone revealed that 15 cases were referred to a surgeon for lumpectomy, with 8 undergoing incision and drainage of the lesion, while the remaining 16 cases opted for conservative management. Adverse effects attributed to CS use were observed in 25 patients (18.4%), leading to treatment cessation in 8 individuals. These adverse reactions included weight gain in 14 cases, high blood sugar in five, proximal myopathy in three, and diarrhea, hemangioma, and hypertension, each occurring once.

Out of the 48 cases who were administered MTX, either as the primary treatment or as an alternative regimen, complete resolution of symptoms was observed in 23 patients (47.9%), while one patient experienced incomplete resolution. Resolution followed by recurrence was noted in 5 cases (10.4%), and 12 patients (25%) showed no discernible improvement. Seven patients discontinued oral MTX, five due to poor compliance, one due to pregnancy, and one due to side effects.

Follow‐up phone calls with patients who discontinued oral MTX showed that six cases opted for conservative management, while one patient was referred to a surgeon for incision and drainage of the lesion.

MTX‐related side effects were reported in three patients (6.2%), with one patient discontinuing the treatment. These side effects included nausea in two patients (4.2%) and an increase in liver enzymes in one patient. Switching from oral MTX to the subcutaneous form resolved the side effects in two patients, but in one case, the side effects were severe enough to warrant discontinuation of the treatment.

In total, 12 patients (8.8%) were administered subcutaneous MTX, with nine cases (75%) experiencing complete resolution of symptoms. However, two patients (16.7%) did not show significant improvement, and one patient (8.3%) discontinued treatment.

Lastly, two patients (1.3%) who did not achieve complete resolution with steroid and MTX treatment underwent surgical lumpectomy. No clinically significant wound‐healing complications were identified during routine follow‐up after surgical excision.

### 3.3. Final Outcome

The final follow‐up was conducted via telephone in December 2022. Patients were questioned about their symptoms, any episodes of relapse, or treatment side effects. Those reporting mastitis symptoms or adverse effects were advised to visit the clinic for further evaluation. The median follow‐up duration was 5 years (IQR, 4–6 years).

By the end of the study, 139 patients (89.1%) reported complete resolution, while 9 patients (5.8%) exhibited incomplete resolution, and 8 cases (5.1%) continued to experience symptoms. The median duration of the disease, determined based on clinical examination of patients, was 18 months (IQR, 7–36), with a range from 2 to 126 months.

### 3.4. Factors Linked to Prolonged Illness Duration

Patients who developed breast abscesses after treatment had a notably longer illness duration compared to those who did not experience such complications (median duration of 23 [IQR, 11–44] months vs. 12 [IQR, 6–30.5] months, *p* = 0.04). No significant associations were found between other characteristics and extended illness duration (see Table 2).

**TABLE 2:** Overview of demographic data, clinical symptoms, and median illness duration among patients diagnosed with lobular granulomatous mastitis (*n* = 156).

Characteristics	Frequency (%)	Median duration of the disease, months (IQR, %25, %75)	*p* value
Demographic features			
Marital status			
Married	150 (96.2%)	18 (7, 36.25)	0.97
Single	6 (3.8%)	18 (9, 35.5)	
History			
Oral contraceptive			
Positive	15 (9.6%)	22 (10, 37)	0.61
Negative		18 (7, 36)	
Family history of breast cancer			
Positive	12 (7.7%)	13 (6, 29.25)	0.40
Negative		18 (7.75, 36.25)	
Pattern of involvement			
Bilateral			
Positive	30 (19.2%)	23 (12, 49.5)	0.07
Negative		14.5 (7, 36)	
Clinical manifestations			
Mass			
Positive	114 (73.1%)	20 (8.25, 36.5)	0.62
Negative		12 (6.75, 32.25)	
Mass > 5 cm on sonography			
Positive	18 (11.5%)	21 (6.75, 37.25)	0.78
Negative		18 (7, 36)	
Abscess			
Positive	43 (27.6%)	22 (11, 48)	0.19
Negative		13 (6, 36)	
Pain			
Positive	102 (65.4%)	18 (9.75, 36.5)	0.30
Negative		12 (6, 35.5)	
Erythema			
Positive	56 (35.9%)	24 (7.5, 41.75)	0.13
Negative		12 (7, 30)	
Nipple retraction			
Positive	41 (26.3%)	24 (9, 51)	0.10
Negative		16.5 (7, 32)	
Discharge			
Positive	42 (26.9%)	22 (9, 44)	0.24
Negative		15 (7, 36)	
Axillary lymphadenopathy			
Positive	27 (17.3%)	24 (12, 48)	0.19
Negative		18 (7, 36)	
Abscess formation following treatment			
Positive	50 (32.1%)	23 (11, 44)	0.04^∗^
Negative		12 (6, 30.5)	
Erythema nodosum			
Positive	8 (5.1%)	24 (13.5, 43.5)	0.42
Negative		18 (7, 36)	

*Note:* The disease duration was defined as the time elapsed between the onset of symptoms and achieving complete recovery without any subsequent relapse.

Abbreviation: IQR, interquartile range.

^∗^ *p* < 0.05 considered statistically significant.

### 3.5. IGM and Hyperprolactinemia/Prolactinoma

Twenty patients (12.8%) exhibited elevated levels of plasma prolactin (normal range 4.8–23.3 ng/mL), with no history of pregnancy or prior medication use. Brain magnetic resonance imaging (MRI) revealed pituitary adenomas in four cases (2.6%). Patients with IGM and hyperprolactinemia experienced a prolonged disease course compared to those with normal plasma prolactin levels (35.5 [IQR, 18.5–60.75] months vs. 13 [IQR, 7–30] months, *p* = 0.001). These patients were treated with bromocriptine or cabergoline, as outlined in Table 3.

**TABLE 3:** Overview of treatment strategies employed in patients with prolactinoma (*n* = 4).

Patient	Plasma prolactin levels (ng/mL)	The course of the disease (months)	MRI findings	Treatment approaches
1	69	48	Prolactinoma	Prednisone, oral, and subcutaneous MTX failed to prevent relapse, leading to the patient being referred to a surgeon for breast lumpectomy. Eventually, remission was attained.
2	112	31	Prolactinoma	Prednisone was initiated, ultimately resulting in remission.
3	114	20	Prolactinoma	Prednisone and oral MTX failed to prevent relapse. Subcutaneous MTX was initiated, ultimately resulting in remission.
4	201	54	Prolactinoma	Prednisone, oral and subcutaneous MTX, as well as surgery (breast lumpectomy), all failed to prevent relapse. Ultimately, repeated incision and drainage of the lesion led to remission.

*Note:* Plasma prolactin levels exceeding 23.3 ng/mL are considered indicative of hyperprolactinemia. SC: subcutaneous.

Abbreviations: MRI, magnetic resonance imaging; MTX, methotrexate.

### 3.6. LGM and Diabetes Mellitus

Out of the 156 patients enrolled in this study, 12 (7.7%) had a medical history of diabetes mellitus. The median duration of symptoms until complete remission in patients with diabetes compared to nondiabetic individuals was 24 months (IQR, 11.25–36.75) versus 18 months (IQR, 7–36), respectively, with no significant difference observed (*p* = 0.61). Two of these patients exhibited elevated plasma prolactin levels attributed to lactation and prolactinoma. Details of the treatment approaches are outlined in Table 4.

**TABLE 4:** Overview of treatment strategies employed in patients with diabetes (*n* = 12).

Patient	FBS (mg/dL)	HgA1C (%)	Course of the disease (months)	Treatment approaches
1	75	—	3	A 72‐year‐old patient underwent expectant management, leading to remission.
2	108	6.1	37	Prednisone was initiated, ultimately resulting in remission. The patient had elevated plasma levels of prolactin.
3	127	7.5	36	Prednisone treatment was commenced, leading to remission.
4	154	—	11	Prednisone (20 mg/day) administration resulted in elevated fasting blood sugar (FBS) levels. The treatment was discontinued, and conservative management was initiated, involving wound drainage with traditional medicine, ultimately resulting in remission.
5	161	5.7	24	Prednisone treatment led to elevated FBS levels. Subsequently, MTX therapy was initiated, resulting in remission.
6	175	7	3.5	Prednisone treatment led to remission.
7	217	9.9	12	Prednisone resulted in elevated FBS levels. MTX therapy was initiated, leading to remission.
8	267	—	60	The patient had a history of LGM and underwent lumpectomy, followed by recurrence during pregnancy, which resolved with expectant management.
9	285	12.1	24	Initially, MTX therapy was initiated, but recurrence occurred despite 2 years of treatment. The therapy was discontinued, and conservative management, involving physical drainage of the wound, was initiated, resulting in remission 10 months later.
10	294	8.6	12	Prednisone treatment led to remission.
11	330	12.1	54	Prednisone treatment led to elevated FBS levels. Subsequently, MTX therapy was initiated, resulting in relapse. Conservative management involved wound incision and drainage, ultimately leading to remission. The patient had a history of prolactinoma.
12	341	12.1	36	MTX therapy was initiated, leading to remission.

*Note:* AB: antibiotic, Hg A1C: hemoglobin A1C, MTX: methotrexate.

Abbreviation: FBS, fasting blood sugar.

### 3.7. LGM and EN

In our study, eight cases (5.1%) presented with both LGM and EN. Managing symptoms in this subgroup posed greater challenges, with half of the patients (four out of eight) having experienced a prior episode of LGM. Additionally, the disease manifestation was more complex, with five patients (62.5%) exhibiting multiple breast masses, and one case (12.5%) involving both breasts. Furthermore, half of the cases (four out of eight) experienced concurrent arthritis and EN alongside LGM.

Among the eight cases receiving steroid treatment for LGM and EN, five cases experienced healing, while two encountered recurrences. For the three patients who showed no improvement with prednisone, and one who experienced recurrence, oral MTX was administered. Out of the four cases receiving oral MTX, two did not show improvement, and one experienced recurrence, prompting all three to switch to subcutaneous MTX. Ultimately, all cases achieved complete recovery.

The disease course in patients with both LGM and EN was observed to be 24 months (IQR, 13.5–43.5), compared to 18 months (IQR, 7–36) in those without EN; however, this difference was not statistically significant (*p* = 0.42).

## 4. Discussion

In the present study, approximately 40% of patients treated with CSs achieved complete remission, and MTX was effective in about half of treated patients. At the final follow‐up, more than 90% of patients with LGM attained complete symptom resolution at a median follow‐up of 5 years, regardless of treatment modality or intolerance to CS or MTX therapy. The final outcome of the study, assessed through long‐term follow‐up, demonstrated a high rate of complete resolution among our patients, underscoring the self‐limiting nature of LGM.

In the present study, among 136 cases treated with CSs, a notable proportion (42%) experienced complete resolution of symptoms, while others faced relapse (11%) or showed no improvement (24%). Additionally, a significant number of patients discontinued CSs due to reasons such as poor compliance, adverse medication effects, or pregnancy. A previous meta‐analysis involving 358 LGM patients treated with oral CSs between January 1, 2010, and December 31, 2015, showed complete remission rates ranging from 30.8% to 100%, with recurrence rates ranging from 0% to 46.2%. The pooled estimates for complete remission and recurrence rates of CSs were 71.8% (95% CI [confidence interval] 67.1%, 76.3%) and 20.9% (95% CI 16.1%, 26.4%), respectively. When oral CSs were combined with surgery, the estimated complete remission and recurrence rates were 94.5% (95% CI 88.9%, 98.3%) and 4% (95% CI 1.5%, 8.4%), respectively [17]. In another systematic review and meta‐analysis covering CSs in LGM involving 559 patients up to May 21, 2019, the recurrence rate in the CS‐only group was 17.7%. The relative risk (RR) and risk difference of recurrence in the steroid‐only group compared with those in the surgery‐only group were 2.99 (95% CI 0.28–31.33) and 0.14 (95% CI − 0.01–0.30), respectively, showing no significance. Additionally, the RR of recurrence in the CS‐only group compared to the combined therapy of CSs plus surgery was 6.13 (95% CI 0.41–81.62), again showing no significance. However, the risk of recurrence in the steroid‐only group was significantly higher than in the CSs plus surgery group (risk difference: 0.28, 95% CI 0.11–0.44) [17]. Discrepancies in outcomes between our patients and previous studies may be attributed to the inclusion of treatment discontinuation as a separate category within treatment outcomes. While some patients who discontinued treatment experienced improvement due to the self‐limiting nature of the disease, they were classified as treatment discontinuation cases rather than being included in the remission group.

Oral MTX is commonly employed as a second‐line therapy for patients with IGM who either do not respond to or cannot tolerate steroids [13]. While MTX showed promising results in half of our patients, approximately 10% experienced recurrences during follow‐up. Moreover, MTX‐related side effects were reported, leading to treatment discontinuation in some other cases. Adverse reactions to MTX have been documented in previous studies, ranging from none to 18.2% [18–20]. However, in our cases, side effects were observed in only about 5% of patients. Retrospective analyses have reported remission rates ranging from 75% to 100% and recurrence rates between 12.5% and 15.8% with MTX monotherapy, typically administered at doses of 7.5–25 mg per week over an average treatment duration of 8.5–15 months [14, 20, 21]. The choice between oral and subcutaneous MTX administration depends on individual patient factors and tolerability. Additionally, previous literature indicates that MTX is often used in conjunction with CSs. Combining prednisone with MTX at doses of 5–10 mg per week has led to remission rates ranging from 58.5% to 100%, with relapse rates varying from zero to 28.6% [18, 19, 21–25].

A small subset of patients in our cohort eventually required surgical intervention, such as lumpectomy. A meta‐analysis revealed that surgical excision significantly increased the complete remission rate compared to steroid therapy (*p* = 0.0003). However, the study reported no significant difference in effectiveness between observation and surgical intervention for early LGM patients with mild symptoms (RR = 0.78, 95% CI [0.55, 1.11], *p* = 0.17) [26]. Another meta‐analysis reported a recurrence rate of 22.5% for various surgical procedures including drainage, excision, and lumpectomy [27]. Moreover, surgical procedures carry potential complications such as impaired wound healing, fistula or abscess formation (ranging from 4.7% to 30.0%), scarring, and breast asymmetry. These factors have led surgical intervention to be considered an alternative approach for patients who do not respond to medical therapy rather than being the primary management option [28–34].

There has been considerable debate regarding the optimal approach to managing LGM, whether it should be actively treated or managed through observation, and the most effective treatment modality for those who require intervention: medical or surgical. LGM is categorized into four stages based on disease progression and clinical presentation [1]: self‐limited stage [2], congestive swelling stage [3], abscess formation stage, and [4] complex refractory stage. At the self‐limited stage, watchful waiting through clinical examination has been proposed as a reasonable strategy. During this stage, symptoms may spontaneously resolve or remain stable for months or even years [35]. In our study, 11 (7%) cases initially underwent expectant management and showed improvement over a median period of approximately 10 months. Similarly, previous research has indicated that in patients who were observed without active treatment, symptom resolution typically occurred within a range of 5–14.5 months [9–11, 36–38]. Davis et al. observed that delayed first childbirth was correlated with a longer duration of watchful waiting [9].

In our investigation, the occurrence of breast abscesses subsequent to treatment was linked to prolonged mastitis duration (median duration: 23 months vs. 12 months in patients without abscesses). This association underscores the clinical significance of abscess development as a marker of disease severity and complexity. Similarly, prior research has shown that the recurrence of LGM is more prevalent among those who develop recurrent abscesses during treatment [39]. Hur et al. demonstrated that patients with lesions measuring 1–2 cm in diameter tended to experience a self‐limited condition, while those with larger lesions (> 5 cm) were more prone to progress to breast abscess [36].

Our study did not identify significant associations between other characteristics and extended illness duration. Characteristics like marital status, the use of hormonal contraceptives, a family history of breast cancer, and clinical signs such as the presence of a mass, erythema, nipple discharge, axillary lymphadenopathy, EN, and the size of the mass did not demonstrate statistically significant associations with the duration of the disease. Previous studies have indicated that purulent nipple discharge, skin lesions, bilateral disease, pain, a body mass index (BMI) of ≥ 24 (indicative of overweight/obesity), and an elevated follicle‐stimulating hormone (FSH)/luteinizing hormone (LH) ratio are associated with a heightened risk of recurrence [40–43]. Our findings demonstrated that although patients experiencing pain, nipple retraction, fistula drainage, and bilateral involvement tended to have a longer disease duration, these differences did not reach statistical significance.

LGM can present unique challenges in individuals with comorbidities such as diabetes mellitus, hyperprolactinemia, and EN. Patients with LGM and hyperprolactinemia experienced a prolonged disease course compared to those with normal prolactin levels, emphasizing the influence of hormonal factors on disease progression. Treatment with bromocriptine or cabergoline was effective in managing hyperprolactinemia‐associated LGM, highlighting the importance of addressing underlying hormonal imbalances in treatment strategies. Consistent with our findings, previous studies have also suggested an association between elevated prolactin levels and the recurrence of LGM [42, 44]. Elevated prolactin levels can lead to increased milk production and accumulation within the mammary lobules, potentially causing infection or extravasation into the perilobular stroma, triggering a T‐cell‐mediated immune response and subsequent granuloma formation [45]. Moreover, prolactin has been shown to activate the NF‐kB signaling pathway in mammary epithelial cells, leading to the production of proinflammatory cytokines such as IL (interleukin)‐1, IL‐6, TNF (tumor necrosis factor)‐a, INF (interferon)‐c, and GM‐CSF (granulocyte macrophage colony stimulating factor), which could further exacerbate inflammation and contribute to granuloma formation in the breast [46]. Thus, elevated prolactin levels may exacerbate inflammation and influence the duration of recovery, underscoring the importance of routine screening for hyperprolactinemia in LGM patients [10, 47].

In our cohort, 12.8% of LGM patients exhibited elevated plasma prolactin levels. A meta‐analysis conducted in 2023 reported a prevalence of hyperprolactinemia in 19.7% of LGM patients (99 out of 502) [27]. Furthermore, 2.6% of our cases were diagnosed with prolactinoma. This finding suggests an unusually high prevalence of prolactinoma among LGM patients compared to the incidence of 60–100 cases per 1,000,000 individuals in general population [48].

Treating LGM in special patient groups, such as those with diabetes, poses notable challenges. Although diabetes has been reported in 6.2% of LGM cases, no clear association has been established between diabetes and the onset or recurrence of LGM [41]. Hyperglycemia is known to result in the formation of glycosylated end products that may stimulate B‐cell proliferation and cytokine release, leading to an autoimmune response in various organs, including the breasts [49]. It is also suggested that persistent hyperglycemia, along with increased intermolecular cross‐linkage and glycosylation, impedes collagen degradation, contributing to connective tissue accumulation in the breasts [50]. In our study, diabetes was identified in 7.7% of cases, yet these patients did not exhibit a prolonged disease course or a higher recurrence rate for LGM. Consistent with our findings, a study on LGM patients scheduled for observation found no significant difference in the time to resolution between diabetic and nondiabetic patients (RR = 0.98, 95% CI [0.59–1.63], *p* = 0.94) [9]. Additionally, another study investigating factors contributing to LGM recurrence did not find a significant association between diabetes and disease recurrence (OR = 1.38, 95% CI [0.59–3.25], *p* = 0.45) [41]. While diabetes did not appear to significantly impact the disease course, careful management of glycemic control and potential interactions with immunosuppressive therapies are warranted in diabetic LGM patients. In our study, five diabetic patients were treated with MTX, resulting in remission for four of them. One patient did not respond to treatment, and recurrence occurred in another patient, although both cases improved with conservative management. Prior studies have also advocated for MTX as the preferred initial treatment for diabetic LGM patients over steroids [20].

We identified that patients with a subtype of LGM that is associated with EN exhibited more complex disease manifestations, including multiple breast masses and concurrent arthritis. Additional research has also indicated that patients with concurrent LGM and EN often exhibit more extensive breast involvement (*p* = 0.01) [51, 52]. The term GMENA (Granulomatous Mastitis, Erythema Nodosum, Arthritis) syndrome was introduced by Parperis et al. in 2021, indicating the simultaneous presence of LGM with EN and arthritis [53]. Both GM and EN show similar histopathological findings with chronic inflammation and granulomas, suggesting a shared underlying cause [53]. Despite the complexity, treatment approaches involving CSs and MTX were effective in achieving complete recovery in this subgroup of patients. Previous studies also have suggested that individuals with LGM and EN may exhibit favorable responses to systemic immunosuppression due to shared pathophysiological mechanisms linked to autoimmunity [54, 55]. For instance, one study involving 11 patients with LGM and EN treated with methylprednisolone reported full recovery within 12 weeks, with no recurrence observed during the 60‐month follow‐up period [56].

In our cohort, patients with LGM and EN who received either prednisone or MTX tended to have a longer disease duration compared to those without EN, although this contrast did not reach statistical significance. However, two previous studies demonstrated a significant association between the presence of both LGM and EN and a prolonged disease course (*p* < 0.001 and *p* = 0.005) [52, 57]. In our study, half of the patients with both LGM and EN had experienced a previous episode of LGM managed with prednisolone. Several studies have indicated a significantly higher recurrence rate in LGM patients with EN compared to those without EN (42.31% vs. 16.00%, *p* < 0.001) [57, 58]. However, another study found a higher recurrence rate in the EN group, but the difference was not statistically significant (16.7% vs. 6.7%, *p* = 0.24) [52].

Our study has limitations that warrant acknowledgment. This study recruited patients exclusively from our institution, which could introduce selection bias. This bias might result in the overrepresentation of individuals with more severe or treatment‐resistant cases of LGM, potentially inflating assessments of disease severity and treatment efficacy. However, the inclusion of a large sample size and patients with various underlying conditions may partially address these limitations.

## 5. Conclusion

Our findings demonstrate that more than 90% of LGM cases achieved improvement over a median follow‐up of 5 years. Our study identifies breast abscess formation as a significant characteristic associated with a prolonged disease course in patients with LGM. Clinicians should be vigilant in monitoring for signs of abscess development and employ timely interventions, if needed, to optimize patient outcomes. Our findings also underscore the importance of considering comorbidities in the management of LGM. Tailored treatment approaches addressing hormonal imbalances, glycemic control, and complex disease manifestations are crucial for optimizing outcomes in LGM patients with diabetes mellitus, hyperprolactinemia, and EN. Further studies exploring the mechanisms underlying these associations are warranted to inform more targeted therapeutic interventions in this complex clinical scenario.

**Nomenclature**
LGM: Lobular granulomatous mastitis
MTX: Methotrexate
IQR: Interquartile range
CSs: Corticosteroids
PCR: Polymerase chain reaction
ACE: Angiotensin‐converting enzyme
FBS: Fasting blood sugar
ESR: Erythrocyte sedimentation rate
EN: Erythema nodosum
CBC: Complete blood count
BMI: Body mass index
FSH: Follicle‐stimulating hormone
LH: Luteinizing hormone
RR: Relative risk
CI: Confidence interval
IL: Interleukin
TNF: Tumor necrosis factor
INF: Interferon
GM‐CSF: Granulocyte macrophage colony stimulating factor
GMENA: Granulomatous mastitis erythema nodosum arthritis

## Author Contributions

Mina AkbariRad: conceptualization. Abdollah Firoozi: conceptualization and revising the manuscript. Fereshte Sheybani: design of the work, analysis and interpretation of data, writing original draft of the manuscript, and revising the manuscript. Samaneh Sajjadi: design of the work. Maryam Emadzadeh: revising the manuscript. Marzieh Kazerani: revising the manuscript. Sajad Ataei Azimi: revising the manuscript. Mahdieh Mottaghi: investigation, creation of software used, writing original draft of the manuscript, and project administration.

## Funding

There was no funding.

## Disclosure

All authors read and approved the final manuscript. This manuscript has been previously submitted as a preprint [59].

## Ethics Statement

This study was supported by the Mashhad University of Medical Sciences (Code: 4010734) and ethically approved by the Ethics Committee of the Mashhad University of Medical Sciences (Code: IR.MUMS.MEDICAL.REC.1401.609).

## Consent

All patients are conscious and consented to the publication of the data.

## Conflicts of Interest

The authors declare no conflicts of interest.

## Data Availability

The data used to support the findings of this study are available from the corresponding author upon request.
